# Antimicrobial Use in Pediatric Oncology and Hematology: Protocol for a Multicenter Point-Prevalence Study With Qualitative Expert Panel Assessment

**DOI:** 10.2196/35774

**Published:** 2022-06-20

**Authors:** Cihan Papan, Katharina Reifenrath, Katharina Last, Andishe Attarbaschi, Norbert Graf, Andreas H Groll, Johannes Huebner, Hans-Jürgen Laws, Thomas Lehrnbecher, Johannes Liese, Luise Martin, Tobias Tenenbaum, Stefan Weichert, Simon Vieth, Ulrich von Both, Markus Hufnagel, Arne Simon

**Affiliations:** 1 Center for Infectious Diseases Institute of Medical Microbiology and Hygiene Saarland University Homburg Germany; 2 Pediatric Hematology and Oncology University Children’s Hospital Saarland University Homburg Germany; 3 Department of Pediatric Hematology and Oncology St. Anna Children's Hospital Medical University of Vienna Vienna Austria; 4 Infectious Disease Research Program Department of Pediatric Hematology/Oncology and Center for Bone Marrow Transplantation University Children's Hospital Münster Münster Germany; 5 Division of Pediatric Infectious Disease Dr v Hauner Children's Hospital University of Munich Munich Germany; 6 Department of Pediatric Oncology Hematology and Clinical Immunology University of Duesseldorf Düsseldorf Germany; 7 Division for Pediatric Hematology and Oncology Hospital for Children and Adolescents, University Hospital Goethe University Frankfurt am Main Frankfurt am Main Germany; 8 Pediatric Infectious Diseases and Immunology University Children's Hospital University of Würzburg Würzburg Germany; 9 Department of Pediatric Respiratory Medicine Immunology and Critical Care Medicine Charité - Universitätsmedizin Berlin Berlin Germany; 10 Pediatric Infectious Diseases Department of Pediatrics, Medical Faculty Mannheim Heidelberg University Mannheim Germany; 11 Department of Pediatrics University Medical Center Schleswig-Holstein, Campus Kiel Kiel Germany; 12 Division of Pediatric Infectious Diseases and Rheumatology Department of Pediatrics and Adolescent Medicine, University Medical Center, Medical Faculty University of Freiburg Freiburg Germany

**Keywords:** point-prevalence study, antimicrobial stewardship, pediatric oncology, pediatric hematology, expert panel, antimicrobial resistance, oncology, cancer, pediatrics

## Abstract

**Background:**

Because infections are a major driver of morbidity and mortality in children with hematologic or oncologic diseases, antimicrobials are frequently prescribed in pediatric oncology practice. However, excess or inappropriate use of antimicrobials is directly linked to the emergence of antimicrobial resistance. Although point-prevalence studies have examined the extent of antimicrobial use, a comprehensive qualitative evaluation of individual antimicrobial prescriptions remains lacking.

**Objective:**

The aim of this study is to identify appropriate versus inappropriate antimicrobial use among pediatric cancer patients in a point-prevalence study, followed by an expert panel adjudication process and a subsequent report of these findings to participating centers. This study also aims to improve the quality of patient care by informing centers about discrepancies between internal standards of care and national guidelines.

**Methods:**

Our point-prevalence study is performed at pediatric cancer centers in Germany and Austria. All patients under 18 years old who are hospitalized at the time of the study are included. As a supplement to the point-prevalence study, an expert panel is qualitatively assessing each of the antimicrobial prescriptions at the participating centers to review local guidelines and compare them with national guidelines.

**Results:**

As of December 2021, the point-prevalence survey has been conducted at 30 sites and expert panel adjudication for qualitative assessment of each antimicrobial use is ongoing. Results of the study are expected in 2022.

**Conclusions:**

This is the first point-prevalence study conducted among pediatric cancer centers with an integrated, multistep, qualitative approach that assesses each antimicrobial prescription. The results of this study will inform possible interventions for internal guidelines and antimicrobial stewardship programs implemented at pediatric cancer centers. In addition, local guidelines will be compared with national guidelines. Furthermore, this study will contribute to the overall integration of antimicrobial stewardship principles and initiatives in pediatric oncology and hematology, thereby improving safety and quality of care for children and adolescents with cancer and blood disorders.

**International Registered Report Identifier (IRRID):**

DERR1-10.2196/35774

## Introduction

Children with oncologic or hematologic diseases are at particularly high risk for severe infections, especially while on chemotherapeutic treatment, radiation, or stem cell transplantation [1,2]. This vulnerability can be attributed to the immunodeficient state caused by the underlying disease as well as by the anticancer treatment. Among children treated in pediatric cancer centers, the high prevalence of and risk for adverse outcomes related to severe bacterial and fungal infections lead to a high level of antimicrobial prescribing [3,4], both for treatment and prophylaxis [5]. These levels are comparable to those in pediatric intensive care units.

Ill-advised, excess use of antimicrobials has been associated with the epidemiological increase of antimicrobial resistance, an imminent global health care threat. Similarly, at the individual level, severe adverse events such as allergic reactions, acute organ toxicity, or *Clostridioides difficile*–related enterocolitis can result from antimicrobial use [6-8].

In general terms, point-prevalence studies (PPSs) are used to monitor the prescription of antimicrobials in a cross-sectional design in a given setting or across different centers. This allows for benchmarking, as well as for the identification of potential targets for antimicrobial stewardship programs (ASPs) [9-12]. Although a PPS may provide a first impression about local antimicrobial use patterns, a critical appraisal of individual indications, choice of antimicrobials, combination therapy, dosing, deescalation, and therapeutic drug monitoring (TDM) issues (ie, an in-depth qualitative assessment) is often hampered by limitations in the PPS data set or by the lack of available time for discussion of individual cases. Consequently, most published PPSs only contain little or no qualitative data and are rather limited to quantitative assessments of antimicrobial use in broader terms. Another limitation of qualitative assessments is the lack of a gold standard in defining treatment approaches in many infectious disease etiologies (with the exception of febrile neutropenia [FN]) in pediatric oncology and hematology [13,14]. One way to address the issue of this missing gold standard is to assemble an expert panel who employ a set of peer-blind adjudicators to determine the most probable etiology based on the available data [15,16]. However, there are scarce data pertaining to the use of expert panels in the determination of infectious disease etiology in pediatric oncology and hematology.

In our study, we will perform a PPS among pediatric oncology and hematology units in Germany and Austria. Specifically, the study employs external expert panels to conduct an incorporated, extensive, qualitative assessment of internal local guidelines and case-based antibacterial and antifungal use. This unique approach may pave the way for the identification of ASP targets in the participating pediatric cancer centers. The aims of the study are to assess antimicrobial prescribing and suitability of antimicrobial treatment at the patient level in pediatric oncology and hematology units, to investigate the extent to which local guidelines may be in line with national guidelines, and to identify potential quality improvement measures for the participating centers.

## Methods

### Study Design

This is a prospective, multicenter, observational PPS of hospitalized children and adolescents from pediatric cancer centers across Germany and Austria. The study assesses antimicrobial prescribing and the suitability of antimicrobial treatment at the patient level, and also investigates the extent to which local guidelines are in line with national guidelines. The study’s goal is to identify potential quality improvement measures for the participating centers.

### Study Population

All university and district hospitals represented within the German Society for Pediatric Oncology and Hematology (Gesellschaft für Pädiatrische Onkologie und Hämatologie [GPOH]) and the German Society for Pediatric Infectious Diseases (Deutsche Gesellschaft für Pädiatrische Infektiologie [DGPI]) were invited to participate. Invitation announcements were communicated via a society newsletter or conveyed by the principal investigators to department heads. Only centers with an existing internal guideline pertinent to antimicrobial treatment in FN were eligible. Inclusion to the study was irrespective of the level of health care and the presence of or affiliation to a university hospital. Only patients hospitalized at the time of the PPS were included.

### Point-Prevalence Survey

The point-prevalence survey was conducted on a select weekday between November 30 and December 4, 2020, or between December 7 and December 11, 2020.

The following general data pertaining to the respective pediatric oncology/hematology unit on the day of the point-prevalence survey were collected: numbers of total beds, hospitalized patients, patients on source isolation (eg, due to colonization or infection with a multidrug-resistant pathogen), patients on protective isolation, and patients on antibacterial and/or antifungal therapy.

In addition, the following data for each patient receiving antibacterial and/or antifungal therapy on the point-prevalence day were collected: age and age group, body weight, height, underlying oncologic/hematologic disease, state of disease (first diagnosis vs relapse), trimethoprim/sulfamethoxazole prophylaxis (yes/no), granulocytopenia (ie, <0.5×10^9^/L), mucositis grade III (according to the World Health Organization oral mucositis grading scale), severe graft-versus-host disease (grade III to IV), subcutaneously tunneled or implanted long-term central venous access device (Broviac, Hickman, or Port), creatinine clearance <50 mL/min per 1.73 m^2^, high risk for fungal infections (defined as acute myeloid leukemia undergoing induction therapy, leukemia relapse/not in remission, allogeneic stem cell transplantation, prolonged neutropenia for ≥10 days and steroid therapy, or graft-versus-host-disease grade III-IV), and colonization with multidrug-resistant organisms (eg, methicillin-resistant *Staphylococcus aureus*; vancomycin-resistant enterococci; multidrug-resistant gram-negative bacteria, including extended-spectrum beta-lactamase producers and carbapenem-resistant bacteria according to the German classification system of multidrug-resistant gram-negative bacteria [17]).

Furthermore, for each antibacterial or antifungal drug, the following data were recorded: type of infectious disease diagnosis (fever without source, skin and soft tissue infection, respiratory tract infection, urinary tract infection, intra-abdominal infection, *C. difficile–*associated disease, postoperative wound infection, bloodstream infection/sepsis, infection of the central nervous system, osteomyelitis/arthritis, other infectious disease diagnosis); generic name of antibacterial or antifungal drug; start date of treatment; route of administration (intravenous vs oral), dosage, number of doses per day versus continuous infusion, indication (therapy, prophylaxis, perioperative prophylaxis, indeterminate); empiric therapy (no known source, no pathogen), calculated therapy (source, no pathogen), or directed therapy (pathogen detected); TDM for aminoglycosides, glycopeptides, triazole antifungals; and if TDM performed, the trough and/or peak levels (choice without additional detail).

Participating centers were asked to provide their center-specific standard-of-care guidelines regarding antimicrobial treatment, with particular attention to the management of FN. The rationale for this approach is to not solely rely on the centers’ purported adherence to guidelines of patient management but rather to verify and scrutinize the content of internal guidance documents concerning their alignment with national guidelines.

### Modified Expert Panel Process

**Figure 1:**
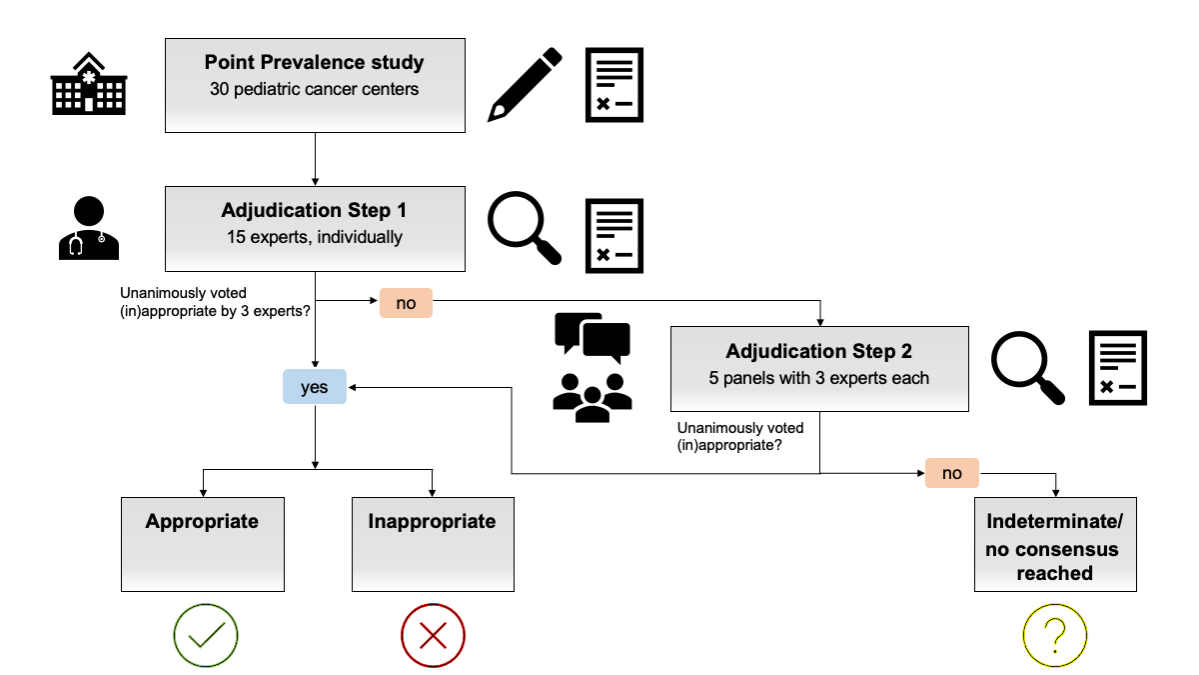
Flow diagram of the point-prevalence study conducted among 30 pediatric cancer centers in Germany and Austria, 2020/2021, and the consecutive multistep adjudication process to define appropriateness of antimicrobial therapy in all reported patients.

In previous studies, an expert panel approach using three independent, blinded adjudicators with field-specific expertise yielded reproducible, consistent results in the determination of infectious disease etiology [15,16]. For this study, we designed a multistep approach to be conducted throughout the year (2021). Before initiating the adjudication process, all data obtained from the point-prevalence survey were deidentified with respect to the participating center. A total of 15 experts from the field of pediatric oncology/hematology and infectious diseases formed five panels with three adjudicators each (Figure 1). Of note, cases from a given center were not assigned to panels that included an adjudicator from that same center. In a first step, while blinded to their panel peers’ adjudications, experts were asked to independently adjudicate each patient’s antimicrobial therapy regarding the suitability of each antimicrobial used. In this process, the adjudicators used both the center-specific local guidelines and the national guidelines for reference in deciding whether the respective therapy was appropriate, inappropriate, or whether appropriateness was indeterminable. When labeled inappropriate, the adjudicators specified the reason for inappropriateness (Table 1). When all three adjudicators unanimously and congruently labeled the antimicrobial use as either appropriate or inappropriate, the case was considered closed. When the adjudicators had differences of opinion, a video conference among the three adjudicators and a moderator was scheduled to reach a consensus by discussing differing perspectives. When consensus could not be reached (eg, because of missing information), the case was classified as “indeterminate.”

**Table 1:** Nonredundant list of items to specify inappropriate therapy as adjudicated by the expert panelists in the point-prevalence study conducted among 30 pediatric cancer centers in Germany and Austria, 2020-2021.

Code	Item	Notes
1	Unnecessary therapy (1)	(Prolonged) antibacterial therapy in patients with respiratory tract infection with viral detection
2	Unnecessary therapy (2)	Prolonged antibacterial therapy (>72 h) in newly diagnosed ALL^a^ with fever without signs of a bacterial infection
3	Inappropriate therapy (1)	No deescalation when pathogen is known
4	Inappropriate therapy (2)	Early escalation (before 48h) without clinical deterioration
5	Dosage	>20% above or below the range for an antimicrobial agent as indicated in the internal guideline
6	Lack of *Pseudomonas aeruginosa* coverage	—^b^
7	Empirical combination therapy	Upfront combination therapy in febrile neutropenia without being founded in the local guideline
8	Primary empirical treatment with meropenem or imipenem	In patients without known colonization with multidrug-resistant organisms
9	Primary empirical treatment with vancomycin or teicoplanin	Exceptions: AML^c^ induction therapy with high-dose cytarabine; extensive skin/soft tissue infection; central line–associated infection; known colonization with MRSA^d^
10	Double coverage (1)	Piperacillin/tazobactam or meropenem, each combined with metronidazole (exception: systemic infection in addition to *Clostridioides difficile* enterocolitis)
11	Double coverage (2)	Meropenem plus aminoglycoside (exceptions: multidrug-resistant *P. aeruginosa*)
12	*C*. *difficile* (1)	Intravenous treatment with vancomycin or teicoplanin in *C*. *difficile*–related enterocolitis
13	*C*. *difficile* (2)	Primary oral treatment with metronidazole instead of vancomycin
14	Surgical antibiotic prophylaxis (1)	>24h without a comprehensible rationale
15	Surgical antibiotic prophylaxis (2)	Drugs of choice: cefazolin, cefuroxime, or ampicillin-sulbactam; in penicillin allergy: clindamycin
16	Bug-drug mismatch	—
17	Aminoglycosides (1)	Application twice or thrice per day instead of once
18	Aminoglycosides (2)	No TDM^e^ performed
19	Aminoglycosides (3)	Contraindications (eg, renal insufficiency)
20	Vancomycin	No TDM performed
21	Linezolid	Twice per day instead of thrice per day in children ≤12 years of age
22	Lack of dosage adjustment in renal insufficiency (creatinine clearance <50 mL·min^–1^·1.72 m^–2^)	for example: ceftazidime, cefepime, ciprofloxacin, imipenem/cilastatin, meropenem, metronidazole, vancomycin
23	Other	Indicated in an open-text format

^a^ALL: acute lymphocytic leukemia.

^b^Not applicable.

^c^AML: acute myeloid leukemia.

^d^MRSA: methicillin-resistant *Staphylococcus aureus*.

^e^TDM: therapeutic drug monitoring.

### Feedback to Centers

A further objective of the study is to improve quality of patient care at the participating centers. This is achieved by reporting back to the centers regarding the panel adjudications, as well as by highlighting discrepancies between local and national guidelines, especially with regard to the center’s internal standards for management of FN and any case-related antimicrobial use deemed to have been inappropriate.

### Outcome Measures

In addition to descriptive data pertaining to the centers and patients receiving antimicrobial therapy, the following outcome parameters are analyzed: antimicrobial prevalence rate per center (number of patients treated with antibacterial and/or antifungal drugs divided by the number of hospitalized patients for a given center on the day of the point-prevalence survey), antimicrobial prevalence rate overall (total number of patients treated with antibacterial and/or antifungal drugs divided by the total number of hospitalized patients for all centers on the day of the point-prevalence survey), rate of appropriate antimicrobial therapies per center and overall, rate of inappropriate antimicrobial therapies with regard to the center-specific standard of care per center and overall, rate of inappropriate antimicrobial therapies with regard to the national guidelines [13,18] per center and overall, rate of indeterminate antimicrobial therapies per center and overall, and rate of antimicrobial therapies without consensus per center and overall.

### Statistical Analysis

Baseline characteristics will be reported descriptively, with counts and percentages used as appropriate. Means with SDs are given for normally distributed data and medians with IQRs are reported for nonnormally distributed data. The primary outcome parameters are reported as point estimates including 95% CIs. Statistical analyses will be performed using SPSS (version 26.0).

### Patient and Public Involvement

No patients are involved in this study protocol.

### Ethics Approval

This study has been approved by the local ethics committee (Ärztekammer des Saarlandes, number 33/20). The need for informed patient consent was waived, since only routinely available data are included in the study, and all patient data were pseudonymized per center.

### Dissemination

The findings of this study will be presented at national and international conferences. Results will be published in international peer-reviewed journals. Reporting will adhere to the STROBE (Strengthening the Reporting of Observational Studies in Epidemiology) guideline for cross-sectional studies [19].

## Results

As of December 2021, the point-prevalence survey has been conducted at 30 sites and expert panel adjudication for qualitative assessment of each antimicrobial use is ongoing. Results of the study are expected for 2022.

## Discussion

To our knowledge, this is the first study to apply a qualitative assessment of antimicrobial use by combining a multistep expert panel approach with a PPS in the context of pediatric oncology and hematology. The aim of this study is to identify appropriate versus inappropriate antimicrobial use, and to subsequently report these findings to the participating centers. In addition, by informing centers about potential discrepancies between internal standards of care and national guidelines [13], the authors of this study also hope to improve quality of patient care.

In contrast to previous studies using expert panel adjudication as a reference standard [16], we provide a modified approach, whereby the blinded panel adjudication is supplemented by a panel discussion with the aim of reaching consensus.

We expect the results of our study to be largely in line with the generally high antimicrobial consumption in pediatric oncology/hematology, given the ample evidence in the existing literature [20,21]. There are limitations to the study design that deserve mentioning. First, while we were able to include many German centers, outreach toward Swiss centers was unsuccessful. However, the study itself remains scalable, and more centers, especially from more countries, could be aimed for in a follow-up study. Another limitation pertains to the patients who were under no antimicrobial treatment, but inappropriately so. The study design did not allow for including these patients who may have been subject to “undertreatment,” which in turn is generally regarded as being less prevalent than overtreatment [15,22]. Moreover, a certain degree of participation bias may have been introduced by centers with an existing ASP who are eager to take part in contrast to centers with little appreciation for the topic, which may coincide with the level of case mix and case complexity. Related to this, an inclusion criterion was the existence of an internal guidance document, which may also raise the possibility of a selection bias. An expert panel should be regarded as an approximation, as imperfect as any other, toward the true etiology of infection in the absence of a gold standard. Some of the known drawbacks of this approach are possible adjudicator fatigue, which may influence outcome classification. In addition, the consensus approach in the second step of the expert panel adjudication, during which adjudicators are no longer blinded but openly discuss each case, may give rise to reciprocal influencing and may favor dominant over reserved adjudicators.

Finally, although the study will inform the participating centers about their guideline adherence and the accuracy of their internal guidance documents, no statements can be made about the sustainability of any change that may be induced by feedback given to the centers.

## Abbreviations

ASP: antimicrobial stewardship program
DGKH: Deutsche Gesellschaft für Krankenhaushygiene
DGPI: Deutsche Gesellschaft für Pädiatrische Infektiologie
ESCMID: European Society of Clinical Microbiology and Infectious Diseases
FN: febrile neutropenia
GPOH: Gesellschaft für Pädiatrische Onkologie und Hämatologie
PPS: point-prevalence study
STROBE: Strengthening the Reporting of Observational Studies in Epidemiology
TDM: therapeutic drug monitoring
